# Differential diagnosis of developmental defects of enamel: a review

**DOI:** 10.1080/07853890.2021.1897305

**Published:** 2021-09-28

**Authors:** Maria Joana Castro, José João Mendes, Susana Vinga, David Casimiro de Andrade

**Affiliations:** a Faculdade de Medicina Dentária da Universidade do Porto (FMUDP), Porto, Portugal; b Centro de Investigação Interdisciplinar Egas Moniz (CiiEM), Instituto Universitário Egas Moniz (IUEM), Caparica, Portugal; c Instituto Superior Técnico da Universidade de Lisboa (IST-UL), Lisboa, Portugal

## Abstract

**Introduction:**

Developmental defects of enamel (D.D.E.) are a change resulting from any disturbance during tooth formation. They may manifest has quantitative defect, hypoplasia, presenting deficit of enamel thickness, or qualitative defect, hypomineralization, which expresses itself as diffuse or unmarked opacity of enamel [1,2]. Their approach and treatment imply a correct diagnosis, so it is essential to distinguish between them and know how to discern from other clinical entities.

**Materials and methods:**

PubMed search with MeSH "enamel hypoplasia" and “differential diagnosis” with the limits: abstract available, humans and 2000-2019. After selecting 19 articles, other PubMed searches were made with: “molar incisor hypomineralization”, “dental fluorosis”, “amelogenesis imperfecta”, “white spot lesions”, “dental erosion” and “attrition”, combined with “differential diagnosis” and same limits. 49 articles were selected. The exclusion criteria were non-existent full-article and articles repeated.

**Results:**

D.D.E. manifest in 3 forms either isolated or in combination: Demarcated opacity: white or discoloured area of enamel, well demarcated from the sound smooth enamel of normal thickness; Diffuse opacity: abnormality translucence of normal thickness enamel without defined margins that manifests as patchy, irregular or cloudy areas that follow the pattern of the perikymata; Hypoplasia: areas of reduced thickness of enamel that can take the form of pits, grooves until complete absence of it [1]. D.D.E. may affect one tooth, a set or all of them [3]. They may affect temporary or permanent teeth or both [2–6]. According D.D.E.’s manifestation and associated aetiology we can distinguish different clinical entities: hypoplasia [2,7–10]; diffuse opacity [7,9]; demarcated opacity [7]; fluorosis [2,8,11]; hypomineralization of 2nd primary molar [7,9]; molar-incisor hypomineralization [5,7–11]; amelogenesis imperfecta [5,7–10]; and D.D.E. associated with inherited systemic disorders [3,4,6]. The D.D.E. result as pre-eruptive lesion to the tooth [11]. Thus, if the lesion occurs post-eruptively, we should admit other clinical entities such as white spot lesion [2,7–9,11] or tooth wear (erosion or attrition) [12].

**Discussion and conclusions:**

D.D.E. exhibit different topographical forms and each call for specific therapeutic approach. Thus, its diagnosis is essential to establish the proper treatment. Furthermore, they may have a significant impact on oral health. They are risk factors of dental caries [1,4,6,8] and erosion [5,9]. They may alter occlusal functions [5], cause of hypersensitivity [5,6,8,9] and compromise aesthetics [5,6,9]. The patient’s cooperation may be impaired because of difficulties to anesthetize [8,9] and repeated adhesion failure of restorative materials [5,8].
